# Supplementary material for: Characteristics and predictors of out-of-hospital cardiac arrest in young adults hospitalized with acute coronary syndrome: A retrospective cohort study of 30,000 patients in the Gulf region
Source: PLoS One. 2023 May 25;18(5):e0286084. doi: 10.1371/journal.pone.0286084 (PMC10212072; doi:10.1371/journal.pone.0286084)
Supplement: S1 Table — ACS: Acute coronary syndrome. Gulf COAST: Gulf Locals with Acute Coronary Syndrome Events Registry. Gulf RACE: Gulf Registry of Acute Coronary Events. KSA: Kingdom of Saudi Arabia. Kuwait REPERFUSE: Reperfusion in ST-Segment–Elevation Myocardial Infarction. NSTEMI: non–ST-segment–elevation myocardial infarction. SPACE: Saudi Project for Assessment of Coronary Events. STARS: Saudi Acute Myocardial Infarction Registry. STEMI: ST-segment–elevation myocardial infarction. UA: Unstable angina. UAE: United Arab Emirates. (DOCX) [file pone.0286084.s001.docx]

**S1 Table: Details of the registries included in the study**

|  | Gulf RACE^7^ | Gulf RACE-2^8^ | Gulf RACE-3^9^ | Gulf COAST^10^ | STARS^11^ | SPACE^12^ | Kuwait REPERFUSE^13^ |
| --- | --- | --- | --- | --- | --- | --- | --- |
| ACS patients, n (%) | 8176/31,620  (25.8) | 7930/31,620  (25) | 2928/31,620  (9.2) | 4061/31,620  (12.8) | 2233/31,620  (7) | 5055/31,620  (15.9) | 1237/31,620  (3.9) |
| No. of hospitals | 64 | 65 | 36 | 29 | 50 | 17 | 7 |
| Countries | Kuwait, Qatar, Bahrain, UAE, Oman, Yemen | KSA, Qatar, Bahrain, UAE, Oman, Yemen | KSA, Kuwait, Qatar, Bahrain, UAE, Oman | Kuwait, Bahrain, UAE, Oman | KSA | KSA | Kuwait |
| Baseline recruitment period (months) | 2006–2007 (6) | 2008–2009 (9) | 2014–2015 (12) | 2012–2013 (12) | 2015–2017 (21) | 2005–2007 (24) | 2014–2015 (12) |
| STEMI patients (N=15532), n (%) | 3200 (20.6) | 3613 (23.2) | 2928 (18.8) | 1015  (6.5) | 1471  (9.4) | 2097 (13.5) | 1208  (7.7) |
| 1-month follow-up, n (%) | No | 3033 (83.9) | No | 981  (96.6) | 888  (60.3) | No | 1208  (100) |
| 1-year follow-up, n (%) | No | 2780 (76.9) | No | 925  (91.1) | 716  (48.6) | No | 1176  (97.3) |

Adapted from Shehab et al.^17^

ACS: Acute coronary syndrome. Gulf COAST: Gulf Locals with Acute Coronary Syndrome Events Registry. Gulf RACE: Gulf Registry of Acute Coronary Events. KSA: Kingdom of Saudi Arabia. Kuwait REPERFUSE: Reperfusion in ST-Segment–Elevation Myocardial Infarction. NSTEMI: non–ST-segment–elevation myocardial infarction. SPACE: Saudi Project for Assessment of Coronary Events. STARS: Saudi Acute Myocardial Infarction Registry. STEMI: ST-segment–elevation myocardial infarction. UA: Unstable angina. UAE: United Arab Emirates.
