# Supplementary material for: Characteristics and predictors of out-of-hospital cardiac arrest in young adults hospitalized with acute coronary syndrome: A retrospective cohort study of 30,000 patients in the Gulf region
Source: PLoS One. 2023 May 25;18(5):e0286084. doi: 10.1371/journal.pone.0286084 (PMC10212072; doi:10.1371/journal.pone.0286084)
Supplement: S2 Table — S2A Table. Characteristics of patients with out-of-hospital cardiac arrest [very young (<40 years) vs older adults]. ES: Effect size. MI: Myocardial infarction. STEMI: ST elevation myocardial infarction. NSTEMI: Non-ST elevation myocardial infarction. LV: Left ventricle. PCI: Percutaneous coronary intervention. CABG: Coronary artery bypass graft surgery. UH: Unfractionated heparin. LWMH: Low molecular weight heparin. *P values were the result of the comparison between very young (<40 years) vs older adults. S2B Table. Characteristics of very young (<40 years) adults (with vs without out-of-hospital cardiac arrest). ES: Effect size. MI: Myocardial infarction. STEMI: ST elevation myocardial infarction. NSTEMI: Non-ST elevation myocardial infarction. LV: Left ventricle. PCI: Percutaneous coronary intervention. CABG: Coronary artery bypass graft surgery. UH: Unfractionated heparin. LWMH: Low molecular weight heparin. (DOCX) [file pone.0286084.s002.docx]

| **S2A Table:** **Characteristics of patients with out-of-hospital cardiac arrest [very young (<40 years) vs older adults].** | | | | | | | | | | |
| --- | --- | --- | --- | --- | --- | --- | --- | --- | --- | --- |
| **Variables** | | **Very young**  **(N=** **54)** | **Old**  **(N=** **557)** | | | **Total**  **(N=611)** | **P value*** | | | **ES** |
| **Demographics** | |  |  | | |  |  | | |  |
| Age | | 35 ± 5 | 60 ± 12 | | | 58 ± 14 | <.0001 | | | 2.21201 |
| Sex (male) | | 53/54  (98.15%) | 436/557  (78.28%) | | | 489/611  (80.03%) | 0.0005 | | | -0.1411 |
| Body mass index | | 27 ± 5 | 28 ± 6 | | | 28 ± 6 | 0.4653 | | | 0.0957 |
| Ethnicity (Arab) | | 23/54  (42.59%) | 351/557  (63.02%) | | | 374/611  (61.21%) | 0.0098 | | | 0.1231 |
| **Medical history** | |  |  | | |  |  | | |  |
| Diabetes mellitus | | 6/54  (11.11%) | 260/556  (46.76%) | | | 266/610  (43.61%) | <.0001 | | | 0.2042 |
| Hypertension | | 4/54  (7.41%) | 287/557  (51.53%) | | | 291/611  (47.63%) | <.0001 | | | 0.2507 |
| Hyperlipidemia | | 7/54  (12.96%) | 190/548  (34.67%) | | | 197/602  (32.72%) | 0.0012 | | | 0.1322 |
| MI or angina | | 5/54  (9.26%) | 199/557  (35.73%) | | | 204/611  (33.39%) | <.0001 | | | 0.1593 |
| Heart failure | | 0/32  (0%) | 50/312  (16.03%) | | | 50/344  (14.53%) | 0.0143 | | | 0.1321 |
| Chronic renal failure | | 0/53  (0%) | 50/538  (9.29%) | | | 50/591  (8.46%) | 0.0204 | | | 0.0954 |
| Smoking status (current smokers) | | 19/54  (35.19%) | 125/557  (22.44%) | | | 144/611  (23.57%) | <.0001 | | | 0.2561 |
| **Presentation data** | |  |  | | |  |  | | |  |
| Grace score | | 140 ± 35 | 198 ± 55 | | | 195 ± 55 | <.0001 | | | 1.09459 |
| Arrival by ambulance | | 17/54  (31.48%) | 178/555  (32.07%) | | | 195/609  (32.02%) | 0.9292 | | | 0.0036 |
| Presentation Killip class  (Killip class 1) | | 30/53  (56.60%) | 231/557  (41.47%) | | | 261/610  (42.79%) | 0.0527 | | | 0.1123 |
| Type of MI (STEMI) | | 46/54  (85.19%) | 430/556  (77.34%) | | | 476/610  (78.03%) | 0.1836 | | | -0.0538 |
| Cardiac arrest as the sentinel event of CAD | | 9/9  (100.00%) | 81/167  (48.50%) | | | 90/176  (51.14%) | 0.0026 | | | -0.2269 |
| LV function in Echo (normal) | | 11/ 49  (22.45%) | 68/425  (16.00%) | | | 79/474  (16.67%) | 0.2883 | | | 0.0891 |
| **Reperfusion therapy details** | |  |  | | |  |  | | |  |
| Symptoms to hospital arrival time | | 231 ± 354 | 337 ± 719 | | | 325 ± 688 | 0.0980 | | | 0.15299 |
| Primary PCI in STEMI patients | | 15/46  (32.61%) | 109/430  (25.35%) | | | 124/476  (26.05%) | 0.2863 | | | -0.0489 |
| CABG | | 0/49  (0%) | 8/459  (1.74%) | | | 8/508  (1.57%) | 0.3516 | | | 0.0413 |
| STEMI thrombolytic therapy | | 19/45  (42.22%) | 189/420  (45.00%) | | | 208/465  (44.73%) | 0.7217 | | | 0.0165 |
| **In hospital medication** | |  |  | | |  |  | | |  |
| Aspirin | | 51/54  (94.44%) | 521/556  (93.71%) | | | 572/610  (93.77%) | 0.8301 | | | -0.0087 |
| GP 2b/3a inhibitors | | 10/54  (18.52%) | 90/556  (16.19%) | | | 100/610  (16.39%) | 0.6586 | | | -0.0179 |
| Other antiplatelets | | 44/54  (81.48%) | 410/557  (73.61%) | | | 454/611  (74.30%) | 0.2062 | | | -0.0511 |
| Heparins (UH or LMWH) | | 46/54  (85.19%) | 490/556  (88.13%) | | | 536/610  (87.87%) | 0.5269 | | | 0.0256 |
| Beta blockers | | 28/54  (51.85%) | 205/555  (36.94%) | | | 233/609  (38.26%) | 0.0313 | | | -0.0872 |
| ACE-I or ARB | | 25/54  (46.29%) | 223/557  (40.04%) | | | 248/611  (40.59%) | 0.3711 | | | -0.0362 |
| Statin | | 45/54  (83.33%) | 455/556  (81.83%) | | | 500/610  (81.97%) | 0.7845 | | | -0.0111 |
| **In hospital course** | |  |  | | |  |  | | |  |
| Elective PCI | | 6/50  (12.00%) | 35/457  (7.66%) | | | 41/507  (8.09%) | 0.2851 | | | -0.0475 |
| Elective coronary angiogram | | 20/45  (44.44%) | 74/402  (18.41%) | | | 94/447  (21.03%) | <.0001 | | | -0.1922 |
| **In hospital complications** | |  |  | | |  |  | | |  |
| In-hospital heart failure | | 11/54  (20.37%) | 268/557  (48.11%) | | | 279/611  (45.66%) | <.0001 | | | 0.1581 |
| Recurrent MI (In Hospital Infarction/Re-Infarction) | | 0/54  (0%) | 39/557  (7.00%) | | | 39/611  (6.38%) | 0.0445 | | | 0.0813 |
| Stroke | | 0/54  (0%) | 25/556  (4.50%) | | | 25/610  (4.10%) | 0.1116 | | | 0.0644 |
| Major Bleeding | | 2/54  (3.70%) | 29/557  (5.21%) | | | 31/611  (5.07%) | 0.6309 | | | 0.0194 |
| **Mortality** | |  |  | | |  |  | | |  |
| Mortality in-hospital | | 7/54  (12.96%) | 258/557  (46.32%) | | | 265/611  (43.37%) | <.0001 | | | 0.1910 |
| One month mortality | | 2/11  (18.18%) | 127/217  (58.53%) | | | 129/228  (56.58%) | 0.0084 | | | 0.1744 |
| One year mortality | | 2/11  (18.18%) | 126/209  (60.29%) | | | 128/220  (58.18%) | 0.0058 | | | 0.1860 |
| ES: Effect size. MI: Myocardial infarction. STEMI: ST elevation myocardial infarction. NSTEMI: Non-ST elevation myocardial infarction. LV: Left ventricle. PCI: Percutaneous coronary intervention. CABG: Coronary artery bypass graft surgery. UH: Unfractionated heparin. LWMH: Low molecular weight heparin.  *P values were the result of the comparison between very young (<40 years) vs older adults. | | | | | | | | | | |
| **S2B Table: Characteristics of very young (<40 years) adults (with vs without out-of-hospital cardiac arrest)** | | | | | | | | |  | |
| **Variables** | **Yes**  **(N=54)** | | | **No**  **(N=2,873)** | **Total**  **(N=2,927)** | | | **P value** | **ES** | |
| **Demographics** |  | | |  |  | | |  |  | |
| Age | 35 ± 5 | | | 36 ± 5 | 36 ± 5 | | | 0.0627 | -0.2706 | |
| Sex (male) | 53/54  (98.15%) | | | 2647/2873  (92.13%) | 2700/2927 (92.24%) | | | 0.1016 | 0.0303 | |
| Body mass index | 27 ± 5 | | | 28 ± 6 | 28 ± 6 | | | 0.7074 | -0.049 | |
| Ethnicity (Arab) | 23/54  (42.59%) | | | 1407/2873  (48.97%) | 1430/2927  (48.86%) | | | 0.5663 | 0.0197 | |
| **Medical history** |  | | |  |  | | |  |  | |
| Diabetes mellitus | 6/54  (11.11%) | | | 618/2863  (21.59%) | 624/2917  (21.39%) | | | 0.0629 | -0.0344 | |
| Hypertension | 4/54  (7.41%) | | | 677/2861  (23.66%) | 681/2915  (23.36%) | | | 0.0052 | -0.0518 | |
| Hyperlipidemia | 7/54  (12.96%) | | | 606/2726  (22.23%) | 613/2780  (22.05%) | | | 0.1038 | -0.0309 | |
| MI or angina | 5/54  (9.26%) | | | 544/2873  (18.93%) | 549/2927  (18.76%) | | | 0.0711 | -0.0334 | |
| Heart failure | 0/32  (0%) | | | 21/1552  (1.35%) | 21/1584  (1.33%) | | | 0.5077 | -0.0166 | |
| Chronic renal failure | 0/53  (0%) | | | 13/2426  (0.54%) | 13/2479  (0.52%) | | | 0.5931 | -0.0107 | |
| Smoking status (current smokers) | 19/54  (35.19%) | | | 1095/2869  (38.17%) | 1114/2923  (38.11%) | | | <.0001 | 0.1059 | |
| **Presentation data** |  | | |  |  | | |  |  | |
| Grace score | 140 ± 35 | | | 80 ± 27 | 81 ± 27 | | | <.0001 | 2.22645 | |
| Arrival by ambulance | 17/54  (31.48%) | | | 560/2838  (19.73%) | 577/2892  (19.95%) | | | 0.0323 | 0.0398 | |
| Presentation Killip class  (Killip class 1) | 30/53  (56.60%) | | | 2575/2833  (90.89%) | 2605/2886  (90.26%) | | | <.0001 | 0.2162 | |
| Type of MI (STEMI) | 46/54  (85.19%) | | | 1823/2872  (63.47%) | 1869/2926  (63.88%) | | | 0.0010 | 0.0608 | |
| LV function in Echo (normal) | 11/49  (22.45%) | | | 883/2487  (35.50%) | 894/2536  (35.25%) | | | 0.0351 | 0.0582 | |
| **Reperfusion therapy details** |  | | |  |  | | |  |  | |
| Symptoms to hospital arrival time | 231 ± 354 | | | 398 ± 737 | 394 ± 730 | | | 0.0033 | -0.2293 | |
| Primary PCI in STEMI patients | 15/46  (32.61%) | | | 386/1823 (21.17%) | 401/1869  (21.46%) | | | 0.0621 | 0.0432 | |
| CABG | 0/49  (0%) | | | 45/2624  (1.71%) | 45/2673  (1.68%) | | | 0.3552 | -0.0179 | |
| STEMI thrombolytic therapy | 19/45  (42.22%) | | | 981/1781  (55.08%) | 1000/1826  (54.76%) | | | 0.0870 | -0.0401 | |
| **In hospital medication** |  | | |  |  | | |  |  | |
| Aspirin | 51/54  (94.44%) | | | 2843/2869  (99.09%) | 2894/2923  (99.01%) | | | 0.0006 | -0.0632 | |
| GP 2b/3a inhibitors | 10/54  (18.52%) | | | 537/2871  (18.70%) | 547/2925  (18.70%) | | | 0.9723 | -0.0006 | |
| Other antiplatelets | 44/54  (81.48%) | | | 2269/2873  (78.98%) | 2313/2927  (79.02%) | | | 0.6542 | 0.0083 | |
| Heparins (UH or LMWH) | 46/54  (85.19%) | | | 2539/2873  (88.37%) | 2585/2927  (88.32%) | | | 0.4698 | -0.0134 | |
| Beta blockers | 28/54  (51.85%) | | | 2254/2869  (78.56%) | 2282/2923  (78.07%) | | | <.0001 | -0.0869 | |
| ACE-I or ARB | 25/54  (46.30%) | | | 1963/2872  (68.35%) | 1988/2926  (67.94%) | | | 0.0006 | -0.0636 | |
| Statin | 45/54  (83.33%) | | | 2715/2868  (94.67%) | 2760/2922  (94.46%) | | | 0.0003 | -0.0667 | |
| **In hospital course** |  | | |  |  | | |  |  | |
| Elective PCI | 6/50  (12.00%) | | | 324/2690 (12.04%) | 330/2740  (12.04%) | | | 0.9923 | -0.0002 | |
| Elective coronary angiogram | 20/45  (44.44%) | | | 891/2574  (34.62%) | 911/2619  (34.78%) | | | 0.1699 | 0.0268 | |
| **In hospital complications** | |  |  | | |  |  | | |  |
| In-hospital heart failure | | 11/54  (20.37%) | 158/2872  (5.50%) | | | 169/2926  (5.78%) | <.0001 | | | 0.0858 |
| Recurrent MI (In Hospital Infarction/Re-Infarction) | | 0/54  (0%) | 40/2873  (1.39%) | | | 40/2927  (1.37%) | 0.3826 | | | -0.0161 |
| Stroke | | 0/54  (0%) | 8/2868  (0.28%) | | | 8/2922  (0.27%) | 0.6975 | | | -0.0072 |
| Major Bleeding | | 2/54  (3.70%) | 7/2871  (0.24%) | | | 9/2925  (0.31%) | <.0001 | | | 0.0841 |
| **Mortality** |  | | |  |  | | |  |  | |
| Mortality in-hospital | 7/54  (12.96%) | | | 36/2871  (1.46%) | 43/2925  (1.47%) | | | <.0001 | 0.1310 | |
| One month mortality | 2/11  (18.18%) | | | 28/1022  (2.74%) | 30/1033  (2.90%) | | | 0.0024 | 0.0944 | |
| One year mortality | 2/11  (18.18%) | | | 34/919  (3.70%) | 36/930  (3.87%) | | | 0.0133 | 0.0812 | |
| ES: Effect size. MI: Myocardial infarction. STEMI: ST elevation myocardial infarction. NSTEMI: Non-ST elevation myocardial infarction. LV: Left ventricle. PCI: Percutaneous coronary intervention. CABG: Coronary artery bypass graft surgery. UH: Unfractionated heparin. LWMH: Low molecular weight heparin. | | | | | | | | | | |
