# Supplementary material for: Characteristics and predictors of out-of-hospital cardiac arrest in young adults hospitalized with acute coronary syndrome: A retrospective cohort study of 30,000 patients in the Gulf region
Source: PLoS One. 2023 May 25;18(5):e0286084. doi: 10.1371/journal.pone.0286084 (PMC10212072; doi:10.1371/journal.pone.0286084)
Supplement: S3 Table — S3A Table Characteristics of patients with out-of-hospital cardiac arrest (young vs older adults) before 2011. ES: Effect size. MI: Myocardial infarction. STEMI: ST elevation myocardial infarction. NSTEMI: Non-ST elevation myocardial infarction. LV: Left ventricle. PCI: Percutaneous coronary intervention. CABG: Coronary artery bypass graft surgery. UH: Unfractionated heparin. LWMH: Low molecular weight heparin. *P values were the result of the comparison between young vs older adults. S3B Table. Characteristics of young adults (with vs without out-of-hospital cardiac arrest) before 2011. ES: Effect size. MI: Myocardial infarction. STEMI: ST elevation myocardial infarction. NSTEMI: Non-ST elevation myocardial infarction. LV: Left ventricle. PCI: Percutaneous coronary intervention. CABG: Coronary artery bypass graft surgery. UH: Unfractionated heparin. LWMH: Low molecular weight heparin. (DOCX) [file pone.0286084.s003.docx]

| **S3A Table: Characteristics of patients with out-of-hospital cardiac arrest (young vs older adults) before 2011.** | | | | | | | | | | |
| --- | --- | --- | --- | --- | --- | --- | --- | --- | --- | --- |
| **Variables** | | **Young**  **(N=79)** | **Old**  **(N=183)** | | | **Total**  **(N=262)** | **P value*** | | | **ES** |
| **Demographics** | |  |  | | |  |  | | |  |
| Age | | 43 ± 6 | 65 ± 10 | | | 58 ± 13 | <.0001 | | | 2.46625 |
| Sex (male) | | 76/79  (96.20%) | 125/183  (68.31%) | | | 201/262  (76.72%) | <.0001 | | | -0.3029 |
| Body mass index | | 27 ± 7 | 28 ± 6 | | | 28 ± 6 | 0.4221 | | | 0.02294 |
| Ethnicity (Arab) | | 41/79  (51.90%) | 132/183  (72.13%) | | | 173/262  (66.03%) | 0.0015 | | | 0.1961 |
| **Medical history** | |  |  | | |  |  | | |  |
| Diabetes mellitus | | 11/79  (13.92%) | 92/182  (50.55%) | | | 103/261  (39.46%) | <.0001 | | | 0.3443 |
| Hypertension | | 18/79  (22.78%) | 115/183  (62.84%) | | | 133/262  (50.76%) | <.0001 | | | 0.3677 |
| Hyperlipidemia | | 13/77  (16.88%) | 61/176  (34.66%) | | | 74/253  (29.25%) | 0.0042 | | | 0.1798 |
| MI or angina | | 24/79  (30.38%) | 94/183  (51.37%) | | | 118/262  (45.04%) | 0.0017 | | | 0.1936 |
| Heart failure | | 1/15  (6.67%) | 10/32  (31.25%) | | | 11/47  (23.40%) | 0.0635 | | | 0.2707 |
| Chronic renal failure | | 1/74  (1.35%) | 11/168  (6.55%) | | | 12/242  (4.96%) | 0.0862 | | | 0.1103 |
| Smoking status (current smokers) | | 50/79  (63.29%) | 46/183  (25.14%) | | | 96/262  (36.64%) | <.0001 | | | 0.3703 |
| **Presentation data** | |  |  | | |  |  | | |  |
| Grace score | | 155 ± 46 | 219 ± 54 | | | 199 ± 60 | <.0001 | | | 1.23226 |
| Arrival by ambulance | | 24/79  (30.38%) | 52/181  (28.73%) | | | 76/260  (29.23%) | 0.7878 | | | -0.0167 |
| Presentation Killip class  (Killip class 1) | | 41/78  (52.56%) | 61/183  (33.33%) | | | 102/261  (39.08%) | 0.0200 | | | 0.1941 |
| Type of MI (STEMI) | | 67/79  (84.81%) | 117/183  (63.93%) | | | 184/262  (70.23%) | 0.0007 | | | -0.2095 |
| Cardiac arrest as the sentinel event of CAD | | 21/30  (70.00%) | 46/110  (41.82%) | | | 67/140  (47.86%) | 0.0062 | | | -0.2315 |
| LV function in Echo (normal) | | 9/58  (15.52%) | 22/141  (15.60%) | | | 31/199  (15.58%) | 0.0031 | | | 0.2637 |
| **Reperfusion therapy details** | |  |  | | |  |  | | |  |
| Symptoms to hospital arrival time | | 162 ± 218 | 187 ± 239 | | | 177 ± 230 | 0.5339 | | | 0.10685 |
| Primary PCI in STEMI patients | | 9/67  (13.43%) | 6/117  (5.13%) | | | 15/184  (8.15%) | 0.0476 | | | -0.1460 |
| CABG | | 0/76  (0%) | 3/182  (1.65%) | | | 3/258  (1.16%) | 0.2602 | | | 0.0701 |
| STEMI thrombolytic therapy | | 35/62  (56.45%) | 49/113  (43.36%) | | | 84/175  (48.00%) | 0.0974 | | | -0.1253 |
| **In hospital medication** | |  |  | | |  |  | | |  |
| Aspirin | | 69/79  (87.34%) | 164/183  (89.62%) | | | 233/262  (88.93%) | 0.5900 | | | 0.0333 |
| GP 2b/3a inhibitors | | 12/79  (15.19%) | 17/183  (9.29%) | | | 29/262  (11.07%) | 0.1624 | | | -0.0863 |
| Other antiplatelets | | 48/79  (60.76%) | 96/183  (52.46%) | | | 144/262  (54.96%) | 0.2152 | | | -0.0766 |
| Heparins (UH or LMWH) | | 62/79  (78.48%) | 163/183  (89.07%) | | | 225/262  (85.88%) | 0.0239 | | | 0.1396 |
| Beta blockers | | 34/79  (43.04%) | 50/182  (27.47%) | | | 84/261  (32.18%) | 0.0134 | | | -0.1531 |
| ACE-I or ARB | | 36/79  (45.57%) | 62/183  (33.88%) | | | 98/262  (37.40%) | 0.0727 | | | -0.1109 |
| Statin | | 55/79  (69.62%) | 139/183  (75.96%) | | | 194/262  (74.05%) | 0.2830 | | | 0.0663 |
| **In hospital course** | |  |  | | |  |  | | |  |
| Elective PCI | | 10/78  (12.82%) | 8/182  (4.40%) | | | 18/260  (6.92%) | 0.0142 | | | -0.1521 |
| Elective coronary angiogram | | 29/77  (37.66%) | 40/179  (22.35%) | | | 69/256  (26.95%) | 0.0113 | | | -0.1583 |
| **In hospital complications** | |  |  | | |  |  | | |  |
| In-hospital heart failure | | 24/79  (30.38%) | 107/183  (58.47%) | | | 131/262  (50.00%) | <.0001 | | | 0.2578 |
| Recurrent MI (In Hospital Infarction/Re-Infarction) | | 4/79  (5.06%) | 8/183  (4.37%) | | | 12/262  (4.58%) | 0.8059 | | | -0.0152 |
| Stroke | | 1/79  (1.27%) | 11/182  (6.04%) | | | 12/261  (4.60%) | 0.0904 | | | 0.1048 |
| Major Bleeding | | 3/79  (3.80%) | 5/183  (2.73%) | | | 8/262  (3.05%) | 0.6456 | | | -0.0284 |
| **Mortality** | |  |  | | |  |  | | |  |
| Mortality in-hospital | | 21/79  (26.58%) | 93/183  (50.82%) | | | 114/262  (43.51%) | 0.0003 | | | 0.2244 |
| One month mortality | | 5/14  (35.71%) | 26/35  (74.29%) | | | 31/49  (63.27%) | 0.0114 | | | 0.3614 |
| One year mortality | | 5/13  (38.46%) | 28/34  (82.35%) | | | 33/47  (70.21%) | 0.0032 | | | 0.4293 |
| ES: Effect size. MI: Myocardial infarction. STEMI: ST elevation myocardial infarction. NSTEMI: Non-ST elevation myocardial infarction. LV: Left ventricle. PCI: Percutaneous coronary intervention. CABG: Coronary artery bypass graft surgery. UH: Unfractionated heparin. LWMH: Low molecular weight heparin.  *P values were the result of the comparison between young vs older adults. | | | | | | | | | | |
| **S3B Table: Characteristics of young adults (with vs without out-of-hospital cardiac arrest) before 2011.** | | | | | | | | |  | |
| **Variables** | **Yes**  **(N=79)** | | | **No**  **(N=7,231)** | **Total**  **(N=7,310)** | | | **P value** | **ES** | |
| **Demographics** |  | | |  |  | | |  |  | |
| Age | 43 ± 6 | | | 44 ± 6 | 44 ± 6 | | | 0.5931 | -0.0633 | |
| Sex (male) | 76/79  (96.20%) | | | 6323/7231  (87.44%) | 6399/7310 (87.54%) | | | 0.0191 | 0.0274 | |
| Body mass index | 27 ± 7 | | | 27 ± 5 | 27 ± 5 | | | 0.9918 | 0.00121 | |
| Ethnicity (Arab) | 41/79  (51.90%) | | | 3700/7231  (51.17%) | 3741/7310  (51.18%) | | | 0.8973 | 0.0015 | |
| **Medical history** |  | | |  |  | | |  |  | |
| Diabetes mellitus | 11/79  (13.92%) | | | 2356/7187  (32.78%) | 2367/7266  (32.58%) | | | 0.0004 | -0.0417 | |
| Hypertension | 18/79  (22.78%) | | | 2505/7185  (34.86%) | 2523/7264  (34.73%) | | | 0.0249 | -0.0263 | |
| Hyperlipidemia | 13/77  (16.88%) | | | 1870/6668  (28.04%) | 1883/6745  (27.92%) | | | 0.0299 | -0.0264 | |
| MI or angina | 24/79  (30.38%) | | | 2130/7232  (29.45%) | 2154/7311  (29.46%) | | | 0.8573 | 0.0021 | |
| Heart failure | 1/15  (6.67%) | | | 69/2700  (2.56%) | 70/2715  (2.58%) | | | 0.3164 | 0.0192 | |
| Chronic renal failure | 1/74  (1.35%) | | | 47/5597  (0.84%) | 48/5671  (0.85%) | | | 0.6332 | 0.0063 | |
| Smoking status (current smokers) | 50/79  (63.29%) | | | 3212/7220  (44.49%) | 3262/7299  (44.69%) | | | <.0001 | 0.0557 | |
| **Presentation data** |  | | |  |  | | |  |  | |
| Grace score | 155 ± 46 | | | 91 ± 29 | 92 ± 30 | | | <.0001 | 2.21285 | |
| Arrival by ambulance | 24/79  (30.38%) | | | 1315/7076  (18.58%) | 1339/7155  (18.71%) | | | 0.0075 | 0.0316 | |
| Presentation Killip class  (Killip class 1) | 41/78  (52.56%) | | | 6189/7069  (87.55%) | 6230/7147  (87.17%) | | | <.0001 | 0.2036 | |
| Type of MI (STEMI) | 67/79  (84.81%) | | | 3717/7232  (51.40%) | 3784/7311  (51.76%) | | | <.0001 | 0.0691 | |
| LV function in Echo (normal) | 9/58  (15.52%) | | | 2062/6344  (32.50%) | 2071/6402  (32.35%) | | | 0.0006 | 0.0523 | |
| **Reperfusion therapy details** |  | | |  |  | | |  |  | |
| Symptoms to hospital arrival time | 162 ± 218 | | | 271 ± 295 | 269 ± 294 | | | 0.0006 | -0.3716 | |
| Primary PCI in STEMI patients | 9/67  (13.43%) | | | 267/3717 (7.18%) | 276/3784  (7.29%) | | | 0.0512 | 0.0317 | |
| CABG | 0/76  (0%) | | | 181/6986  (2.59%) | 181/7062  (2.56%) | | | 0.1551 | -0.0169 | |
| STEMI thrombolytic therapy | 35/62  (56.45%) | | | 2267/3590  (63.15%) | 2302/3652  (63.03%) | | | 0.2788 | -0.0179 | |
| **In hospital medication** |  | | |  |  | | |  |  | |
| Aspirin | 69/79  (87.34%) | | | 7126/7225  (98.63%) | 7195/7304  (98.51%) | | | <.0001 | -0.0963 | |
| GP 2b/3a inhibitors | 12/79  (15.19%) | | | 1198/7227  (16.58%) | 1210/7306  (16.56%) | | | 0.7416 | -0.0039 | |
| Other antiplatelets | 48/79  (60.76%) | | | 5228/7232  (72.29%) | 5276/7311  (72.17%) | | | 0.0230 | 0.0062 | |
| Heparins (UH or LMWH) | 62/79  (78.48%) | | | 6259/7232  (86.55%) | 6321/7311  (86.46%) | | | 0.0372 | -0.0244 | |
| Beta blockers | 34/79  (43.04%) | | | 5546/7222  (76.79%) | 5580/7301  (76.43%) | | | <.0001 | -0.0823 | |
| ACE-I or ARB | 36/79  (45.57%) | | | 4980/7229  (68.89%) | 5016/7308  (68.64%) | | | <.0001 | -0.0520 | |
| Statin | 55/79  (69.62%) | | | 6744/7224  (93.36%) | 6799/7303  (93.10%) | | | <.0001 | -0.0969 | |
| **In hospital course** |  | | |  |  | | |  |  | |
| Elective PCI | 10/78  (12.82%) | | | 843/7230 (11.66%) | 853/7308  (11.67%) | | | 0.7508 | 0.0037 | |
| Elective coronary angiogram | 29/77  (37.66%) | | | 2724/7216  (37.75%) | 2753/7293  (37.75%) | | | 0.9875 | -0.0002 | |
| **In hospital complications** | |  |  | | |  |  | | |  |
| In-hospital heart failure | | 24/79  (30.38%) | 523/7227  (7.24%) | | | 547/7306  (7.49%) | <.0001 | | | 0.0909 |
| Recurrent MI (In Hospital Infarction/Re-Infarction) | | 4/79  (5.06%) | 130/7228  (1.80%) | | | 134/7307  (1.83%) | 0.0315 | | | 0.0252 |
| Stroke | | 1/79  (1.27%) | 30/7219  (0.42%) | | | 31/7298  (0.42%) | 0.2478 | | | 0.0135 |
| Major Bleeding | | 3/79  (3.80%) | 23/7226  (0.32%) | | | 26/7305  (0.36%) | <.0001 | | | 0.0604 |
| **Mortality** |  | | |  |  | | |  |  | |
| Mortality in-hospital | 21/79  (26.58%) | | | 101/7229  (1.40%) | 122/7308  (1.67%) | | | <.0001 | 0.2033 | |
| One month mortality | 5/14  (35.71%) | | | 89/2306  (3.86%) | 94/2320  (4.05%) | | | <.0001 | 0.1251 | |
| One year mortality | 5/13  (38.46%) | | | 115/2024  (5.68%) | 120/2037  (5.89%) | | | <.0001 | 0.1109 | |
| ES: Effect size. MI: Myocardial infarction. STEMI: ST elevation myocardial infarction. NSTEMI: Non-ST elevation myocardial infarction. LV: Left ventricle. PCI: Percutaneous coronary intervention. CABG: Coronary artery bypass graft surgery. UH: Unfractionated heparin. LWMH: Low molecular weight heparin. | | | | | | | | | | |
