# Supplementary material for: Characteristics and predictors of out-of-hospital cardiac arrest in young adults hospitalized with acute coronary syndrome: A retrospective cohort study of 30,000 patients in the Gulf region
Source: PLoS One. 2023 May 25;18(5):e0286084. doi: 10.1371/journal.pone.0286084 (PMC10212072; doi:10.1371/journal.pone.0286084)
Supplement: S4 Table — S4A Table Characteristics of patients with out-of-hospital cardiac arrest (young vs older adults) after 2011. ES: Effect size. MI: Myocardial infarction. STEMI: ST elevation myocardial infarction. NSTEMI: Non-ST elevation myocardial infarction. LV: Left ventricle. PCI: Percutaneous coronary intervention. CABG: Coronary artery bypass graft surgery. UH: Unfractionated heparin. LWMH: Low molecular weight heparin. *P values were the result of the comparison between young vs older adults. S4B Table. Characteristics of young adults (with vs without out-of-hospital cardiac arrest) after 2011. ES: Effect size. MI: Myocardial infarction. STEMI: ST elevation myocardial infarction. NSTEMI: Non-ST elevation myocardial infarction. LV: Left ventricle. PCI: Percutaneous coronary intervention. CABG: Coronary artery bypass graft surgery. UH: Unfractionated heparin. LWMH: Low molecular weight heparin. (DOCX) [file pone.0286084.s004.docx]

| **S4A Table: Characteristics of patients with out-of-hospital cardiac arrest (young vs older adults) after 2011.** | | | | | | | | | | |
| --- | --- | --- | --- | --- | --- | --- | --- | --- | --- | --- |
| **Variables** | | **Young**  **(N=109)** | **Old**  **(N=240)** | | | **Total**  **(N=349)** | **P value*** | | | **ES** |
| **Demographics** | |  |  | | |  |  | | |  |
| Age | | 42 ± 7 | 65 ± 11 | | | 58 ± 14 | <.0001 | | | 2.37243 |
| Sex (male) | | 106/109  (97.25%) | 182/240  (75.83%) | | | 288/349  (82.52%) | <.0001 | | | -0.2613 |
| Body mass index | | 28 ± 4 | 28 ± 7 | | | 28 ± 6 | 0.2933 | | | 0.10364 |
| Ethnicity (Arab) | | 40/109  (36.70%) | 161/240  (67.08%) | | | 201/349  (57.59%) | <.0001 | | | 0.2937 |
| **Medical history** | |  |  | | |  |  | | |  |
| Diabetes mellitus | | 37/109  (33.94%) | 126/240  (52.50%) | | | 163/349  (46.70%) | 0.0013 | | | 0.1724 |
| Hypertension | | 23/109  (21.10%) | 135/240  (56.25%) | | | 158/349  (45.27%) | <.0001 | | | 0.3273 |
| Hyperlipidemia | | 24/109  (22.02%) | 99/240  (41.25%) | | | 123/349  (35.24%) | 0.0005 | | | 0.1866 |
| MI or angina | | 16/109  (14.68%) | 70/240  (29.17%) | | | 86/349  (24.64%) | 0.0036 | | | 0.1558 |
| Heart failure | | 1/93  (1.08%) | 38/204  (18.63%) | | | 39/297  (13.13%) | <.0001 | | | 0.2410 |
| Chronic renal failure | | 1/109  (0.92%) | 37/240  (15.42%) | | | 38/349  (10.89%) | <.0001 | | | 0.2157 |
| Smoking status (current smokers) | | 15/109  (13.76%) | 33/240  (13.75%) | | | 48/349  (13.75%) | <.0001 | | | 0.4817 |
| **Presentation data** | |  |  | | |  |  | | |  |
| Grace score | | 173 ± 45 | 190 ± 47 | | | 188 ± 46 | 0.2976 | | | 0.35472 |
| Arrival by ambulance | | 31/109  (28.44%) | 88/240  (36.67%) | | | 119/349  (34.10%) | 0.1330 | | | 0.0804 |
| Presentation Killip class  (Killip class 1) | | 58/109  (53.21%) | 101/240  (42.08%) | | | 159/349  (45.56%) | 0.2154 | | | 0.1131 |
| Type of MI (STEMI) | | 105/109  (96.33%) | 187/239  (78.24%) | | | 292/348  (83.91%) | <.0001 | | | -0.2283 |
| Cardiac arrest as the sentinel event of CAD | | 8/11  (72.73%) | 15/25  (60.00%) | | | 23/36  (63.89%) | 0.4640 | | | -0.1221 |
| LV function in Echo (normal) | | 14/99  (14.14%) | 34/176  (19.32%) | | | 48/275  (17.45%) | 0.2474 | | | 0.1226 |
| **Reperfusion therapy details** | |  |  | | |  |  | | |  |
| Symptoms to hospital arrival time | | 251 ± 355 | 473 ± 967 | | | 393 ± 808 | 0.0050 | | | 0.27584 |
| Primary PCI in STEMI patients | | 47/105  (44.76%) | 62/187  (33.16%) | | | 109/292  (37.33%) | 0.0491 | | | -0.1152 |
| CABG | | 1/98  (1.02%) | 4/152  (2.63%) | | | 5/250  (2.00%) | 0.3744 | | | 0.0562 |
| STEMI thrombolytic therapy | | 46/105  (43.81%) | 78/185  (42.16%) | | | 124/290  (42.76%) | 0.7852 | | | -0.0160 |
| **In hospital medication** | |  |  | | |  |  | | |  |
| Aspirin | | 108/109  (99.08%) | 231/239  (96.65%) | | | 339/348  (97.41%) | 0.1853 | | | -0.0710 |
| GP 2b/3a inhibitors | | 28/109  (25.69%) | 43/239  (17.99%) | | | 71/348  (20.40%) | 0.0984 | | | -0.0886 |
| Other antiplatelets | | 105/109  (96.33%) | 205/240  (85.42%) | | | 310/349  (88.83%) | 0.0027 | | | -0.1605 |
| Heparins (UH or LMWH) | | 100/109  (91.74%) | 211/239  (88.28%) | | | 311/348  (89.37%) | 0.3317 | | | -0.0520 |
| Beta blockers | | 54/109  (49.54%) | 95/239  (39.75%) | | | 149/348  (42.82%) | 0.0868 | | | -0.0918 |
| ACE-I or ARB | | 49/109  (44.95%) | 101/ 240  (42.08%) | | | 150/349  (42.98%) | 0.6156 | | | -0.0269 |
| Statin | | 95/109  (87.16%) | 211/239  (88.28%) | | | 306/348  (87.93%) | 0.7644 | | | 0.0161 |
| **In hospital course** | |  |  | | |  |  | | |  |
| Elective PCI | | 8/99  (8.08%) | 15/148  (10.14%) | | | 23/247  (9.31%) | 0.5861 | | | 0.0346 |
| Elective coronary angiogram | | 11/83  (13.25%) | 14/108  (12.96%) | | | 25/191  (13.09%) | 0.9530 | | | -0.0043 |
| **In hospital complications** | |  |  | | |  |  | | |  |
| In-hospital heart failure | | 29/109  (26.61%) | 119/240  (49.58%) | | | 148/349  (42.41%) | <.0001 | | | 0.2155 |
| Recurrent MI (In Hospital Infarction/Re-Infarction) | | 5/109  (4.59%) | 22/240  (9.17%) | | | 27/349  (7.74%) | 0.1378 | | | 0.0794 |
| Stroke | | 3/109  (2.75%) | 10/240  (4.17%) | | | 13/349  (3.72%) | 0.5179 | | | 0.0346 |
| Major Bleeding | | 8/109  (7.34%) | 15/240  (6.25%) | | | 23/349  (6.59%) | 0.7038 | | | -0.0203 |
| **Mortality** | |  |  | | |  |  | | |  |
| Mortality in-hospital | | 18/109  (16.51%) | 133/240  (55.42%) | | | 151/349  (43.27%) | <.0001 | | | 0.3639 |
| One month mortality | | 6/34  (17.65%) | 92/145  (63.45%) | | | 98/179  (54.75%) | <.0001 | | | 0.3609 |
| One year mortality | | 4/31  (12.90%) | 91/142  (64.08%) | | | 95/173  (54.91%) | <.0001 | | | 0.3945 |
| ES: Effect size. MI: Myocardial infarction. STEMI: ST elevation myocardial infarction. NSTEMI: Non-ST elevation myocardial infarction. LV: Left ventricle. PCI: Percutaneous coronary intervention. CABG: Coronary artery bypass graft surgery. UH: Unfractionated heparin. LWMH: Low molecular weight heparin.  *P values were the result of the comparison between young vs older adults. | | | | | | | | | | |
| **S4B Table: Characteristics of young adults (with vs without out-of-hospital cardiac arrest) after 2011.** | | | | | | | | |  | |
| **Variables** | **Yes**  **(N=109)** | | | **No**  **(N=3,428)** | **Total**  **(N=3,537)** | | | **P value** | **ES** | |
| **Demographics** |  | | |  |  | | |  |  | |
| Age | 42 ± 7 | | | 43 ± 6 | 43 ± 6 | | | 0.2504 | -0.1265 | |
| Sex (male) | 106/109  (97.25%) | | | 3125/3428  (91.16%) | 3231/3537 (91.35%) | | | 0.0261 | 0.0374 | |
| Body mass index | 28 ± 4 | | | 28 ± 6 | 28 ± 6 | | | 0.0605 | -0.1169 | |
| Ethnicity (Arab) | 40/109  (36.70%) | | | 1748/3428  (50.99%) | 1788/3537  (50.55%) | | | 0.0133 | 0.0494 | |
| **Medical history** |  | | |  |  | | |  |  | |
| Diabetes mellitus | 37/109  (33.94%) | | | 1157/3428  (33.75%) | 1194/3537  (33.76%) | | | 0.9665 | 0.0007 | |
| Hypertension | 23/109  (21.10%) | | | 1135/3428  (33.11%) | 1158/3537  (32.74%) | | | 0.0085 | -0.0442 | |
| Hyperlipidemia | 24/109  (22.02%) | | | 1035/3428  (30.19%) | 1059/3537  (29.94%) | | | 0.0666 | -0.0308 | |
| MI or angina | 16/109  (14.68%) | | | 598/3428  (17.44%) | 614/3537  (17.36%) | | | 0.4530 | -0.0126 | |
| Heart failure | 1/93  (1.08%) | | | 56/2934  (1.91%) | 57/3027  (1.88%) | | | 0.5605 | -0.0106 | |
| Chronic renal failure | 1/109  (0.92%) | | | 41/3428  (1.20%) | 42/3537  (1.19%) | | | 0.7915 | -0.0044 | |
| Smoking status (current smokers) | 15/109  (13.76%) | | | 687/3428  (20.04%) | 702/3537  (19.85%) | | | <.0001 | 0.0863 | |
| **Presentation data** |  | | |  |  | | |  |  | |
| Grace score | 173 ± 45 | | | 93 ± 29 | 94 ± 30 | | | 0.0003 | 2.74124 | |
| Arrival by ambulance | 31/109  (28.44%) | | | 751/3427  (21.91%) | 782/3536  (22.12%) | | | 0.1060 | 0.0272 | |
| Presentation Killip class  (Killip class 1) | 58/109  (53.21%) | | | 3130/3428  (91.31%) | 3188/3537  (90.13%) | | | <.0001 | 0.2938 | |
| Type of MI (STEMI) | 105/109  (96.33%) | | | 2596/3421  (75.88%) | 2701/3530  (76.52%) | | | <.0001 | 0.0834 | |
| LV function in Echo (normal) | 14/99  (14.14%) | | | 1134/2849  (39.80%) | 1148/2948  (38.94%) | | | <.0001 | 0.1668 | |
| **Reperfusion therapy details** |  | | |  |  | | |  |  | |
| Symptoms to hospital arrival time | 251 ± 355 | | | 503 ± 939 | 494 ± 925 | | | <.0001 | -0.2728 | |
| Primary PCI in STEMI patients | 47/105  (44.76%) | | | 1095/2596 (42.18%) | 1142/2701  (42.28%) | | | 0.5996 | 0.0101 | |
| CABG | 1/98  (1.02%) | | | 54/2756  (1.96%) | 55/2854  (1.93%) | | | 0.5064 | -0.0124 | |
| STEMI thrombolytic therapy | 46/105  (43.81%) | | | 1159/2618  (44.27%) | 1205/2723  (44.25%) | | | 0.9257 | -0.0018 | |
| **In hospital medication** |  | | |  |  | | |  |  | |
| Aspirin | 108/109  (99.08%) | | | 3397/3426  (99.15%) | 3505/3535  (99.15%) | | | 0.9366 | -0.0539 | |
| GP 2b/3a inhibitors | 28/109  (25.69%) | | | 763/3426  (22.27%) | 791/3535  (22.38%) | | | 0.3994 | 0.0142 | |
| Other antiplatelets | 105/109  (96.33%) | | | 3242/3428  (94.57%) | 3347/3537  (94.63%) | | | 0.4234 | 0.0135 | |
| Heparins (UH or LMWH) | 100/109  (91.74%) | | | 3136/3426  (91.54%) | 3236/3535  (91.54%) | | | 0.9388 | 0.0013 | |
| Beta blockers | 54/109  (49.54%) | | | 2750/3424  (80.32%) | 2804/3533  (79.37%) | | | <.0001 | -0.1315 | |
| ACE-I or ARB | 49/109  (44.95%) | | | 2554/3428  (74.50%) | 2603/3537  (73.59%) | | | <.0001 | -0.1158 | |
| Statin | 95/109  (87.16%) | | | 3335/3426  (97.34%) | 3430/3535  (97.03%) | | | <.0001 | -0.1037 | |
| **In hospital course** |  | | |  |  | | |  |  | |
| Elective PCI | 8/99  (8.08%) | | | 375/2687 (13.96%) | 383/2786  (13.75%) | | | 0.0955 | -0.0316 | |
| Elective coronary angiogram | 11/83  (13.25%) | | | 569/2227  (25.55%) | 580/2310  (25.11%) | | | 0.0112 | -0.0528 | |
| **In hospital complications** | |  |  | | |  |  | | |  |
| In-hospital heart failure | | 29/109  (26.61%) | 206/3422  (6.02%) | | | 235/3531  (6.66%) | <.0001 | | | 0.1429 |
| Recurrent MI (In Hospital Infarction/Re-Infarction) | | 5/109  (4.59%) | 40/3422  (1.17%) | | | 45/3531  (1.27%) | 0.0017 | | | 0.0527 |
| Stroke | | 3/109  (2.75%) | 13/3412  (0.38%) | | | 16/3521  (0.45%) | 0.0003 | | | 0.0611 |
| Major Bleeding | | 8/109  (7.34%) | 13/3428  (0.38%) | | | 21/3537  (0.59%) | <.0001 | | | 0.1566 |
| **Mortality** |  | | |  |  | | |  |  | |
| Mortality in-hospital | 18/109  (16.51%) | | | 53/3428  (1.55%) | 71/3537  (2.01%) | | | <.0001 | 0.1844 | |
| One month mortality | 6/34  (17.65%) | | | 20/1738  (1.15%) | 26/1772  (1.47%) | | | <.0001 | 0.1882 | |
| One year mortality | 4/31  (12.90%) | | | 33/1637  (2.02%) | 37/1668  (4.24%) | | | <.0001 | 0.0998 | |
| ES: Effect size. MI: Myocardial infarction. STEMI: ST elevation myocardial infarction. NSTEMI: Non-ST elevation myocardial infarction. LV: Left ventricle. PCI: Percutaneous coronary intervention. CABG: Coronary artery bypass graft surgery. UH: Unfractionated heparin. LWMH: Low molecular weight heparin. | | | | | | | | | | |
