# Supplementary material for: Characteristics and predictors of out-of-hospital cardiac arrest in young adults hospitalized with acute coronary syndrome: A retrospective cohort study of 30,000 patients in the Gulf region
Source: PLoS One. 2023 May 25;18(5):e0286084. doi: 10.1371/journal.pone.0286084 (PMC10212072; doi:10.1371/journal.pone.0286084)
Supplement: S5 Table — S5A Table Univariate and multivariate logistic regression for predictors of in-hospital mortality in all ACS patients. OR: Odds ratio. CI: Confidence interval. MI: Myocardial infarction. STEMI: ST elevation myocardial infarction. LV: Left Ventricle. UH: Unfractionated heparin. LWMH: Low molecular weight heparin. OHCA: Out of hospital cardiac arrest. *Interaction analysis was performed for the variables “young age” and “OHCA” (Young*OHCA). S5B Table. Univariate and multivariate logistic regression for predictors of 1-year mortality in all ACS patients. OR: Odds ratio. CI: Confidence interval. MI: Myocardial infarction. STEMI: ST elevation myocardial infarction. LV: Left Ventricle. UH: Unfractionated heparin. LWMH: Low molecular weight heparin. OHCA: Out of hospital cardiac arrest. *Interaction analysis was performed for the variables “young age” and “OHCA” (Young*OHCA). S5C Table. Comparison of mortality rates of OHCA patients before and after 2011. OHCA: Out of hospital cardiac arrest. (DOCX) [file pone.0286084.s005.docx]

**S5A Table:** **Univariate and multivariate logistic regression for predictors of in-hospital mortality in all ACS patients.**

|  | Univariant | Multivariant | | |
| --- | --- | --- | --- | --- |
| Variables | P value | P value | OR | 95% CI |
| **Demographics** |  |  |  |  |
| Sex (Male) | <.0001 | **0.0093** | **0.678** | **0.506–0.909** |
| Ethnicity (Arab) | <.0001 | 0.6745 | 1.060 | 0.809–1.388 |
| **Medical history** |  |  |  |  |
| Diabetes mellitus | <.0001 | 0.3875 | 1.116 | 0.870–1.433 |
| Hypertension | 0.0441 | 0.6500 | 1.061 | 0.821–1.372 |
| Hyperlipidemia | 0.0015 | 0.2475 | 0.852 | 0.649–1.118 |
| History of stroke | <.0001 | 0.6954 | 0.905 | 0.549–1.491 |
| Heart failure | <.0001 | 0.3387 | 1.260 | 0.785–2.021 |
| History of MI or angina | 0.0532 | 0.7156 | 0.947 | 0.707–1.268 |
| Chronic renal failure | <.0001 | 0.5571 | 1.175 | 0.686–2.010 |
| Smoking | <.0001 | 0.1015 | 1.303 | 0.949–1.790 |
| **Presentation data** |  |  |  |  |
| Arrival by ambulance | <.0001 | 0.0783 | 1.277 | 0.973–1.675 |
| Type of MI (STEMI) | <.0001 | 0.1522 | 1.487 | 0.864–2.561 |
| LV function in Echo (normal) | <.0001 | 0.4611 | 1.134 | 0.811–1.586 |
| Presentation Killip class  (Killip class 1) | **<.0001** | **0.0029** | **1.522** | **1.155–2.006** |
| Symptoms to hospital arrival time | 0.0018 | 0.4681 | 1.000 | 1.000–1.000 |
| Revascularization | **<.0001** | **<.0001** | **0.329** | **0.241–0.448** |
| **In hospital medication** |  |  |  |  |
| Aspirin | <.0001 | 0.1638 | 0.563 | 0.251–1.264 |
| GP 2b/3a inhibitors | 0.0072 | 0.7632 | 1.057 | 0.738–1.513 |
| Other antiplatelets | 0.0005 | 0.1526 | 0.770 | 0.538–1.102 |
| Beta blockers | **<.0001** | **0.0004** | **0.630** | **0.488–0.813** |
| ACE-I or ARB | **<.0001** | **<.0001** | **0.418** | **0.323–0.542** |
| Statin | **<.0001** | **0.0022** | **0.499** | **0.320–0.779** |
| **Complications** |  |  |  |  |
| In-hospital heart failure | **<.0001** | **<.0001** | **4.310** | **3.280–5.665** |
| Recurrent MI (In Hospital Infarction/Re-Infarction) | <.0001 | **<.0001** | **2.947** | **1.927–4.506** |
| Stroke | <.0001 | **<.0001** | **4.065** | **2.076–7.959** |
| Major Bleeding | <.0001 | 0.0791 | 1.796 | 0.934–3.451 |
| Atrial Fibrillation | <.0001 | **0.0099** | **1.888** | **1.165–3.061** |
| OHCA | **<.0001** | **<.0001** |  |  |
| Young | **<.0001** | **<.0001** |  |  |
| **Interaction Terms*** |  | 0.6960 |  |  |
| OHCA in Young adults |  |  | 2.673 | 1.271–5.620 |
| OHCA in Older adults |  |  | 3.194 | 1.872–5.450 |
| Young adults with OHCA |  |  | 0.419 | 0.179–0.983 |
| Young adults with no OHCA |  |  | 0.501 | 0.369–0.679 |

OR: Odds ratio. CI: Confidence interval. MI: Myocardial infarction. STEMI: ST elevation myocardial infarction. LV: Left Ventricle. UH: Unfractionated heparin. LWMH: Low molecular weight heparin. OHCA: Out of hospital cardiac arrest.

*Interaction analysis was performed for the variables “young age” and “OHCA” (Young*OHCA).

**S5B Table: Univariate and multivariate logistic regression for predictors of 1-year mortality in all ACS patients.**

| Variables | Univariant | Multivariant | | |
| --- | --- | --- | --- | --- |
|  | P value | P value | OR | 95% CI |
| **Demographics** |  |  |  |  |
| Sex (Male) | <.0001 | 0.5893 | 1.111 | 0.758– 1.630 |
| Ethnicity (Arab) | **<.0001** | **0.0007** | **2.195** | **1.391– 3.463** |
| **Medical history** |  |  |  |  |
| Diabetes mellitus | 0.0011 | 0.3501 | 1.158 | 0.851– 1.576 |
| Hypertension | 0.0055 | 0.5328 | 0.906 | 0.664– 1.236 |
| History of stroke | <.0001 | 0.7976 | 0.918 | 0.476– 1.768 |
| Heart failure | <.0001 | 0.7572 | 1.112 | 0.568– 2.178 |
| History of MI or angina | <.0001 | 0.3323 | 1.176 | 0.848– 1.630 |
| Chronic renal failure | <.0001 | 0.8874 | 0.942 | 0.411– 2.159 |
| Smoking | 0.0001 | 0.3722 | 0.861 | 0.620– 1.196 |
| **Presentation data** |  |  |  |  |
| Arrival by ambulance | 0.0027 | 0.6008 | 1.102 | 0.765– 1.589 |
| Type of MI (STEMI) | 0.0001 | 0.4826 | 1.232 | 0.688– 2.204 |
| LV function in Echo (normal) | <.0001 | 0.9926 | 1.002 | 0.688– 1.458 |
| Presentation Killip class  (Killip class 1) | **<.0001** | **0.0130** | **1.554** | **1.097– 2.201** |
| Symptoms to hospital arrival time | 0.0493 | 0.9733 | 1.000 | 1.000– 1.000 |
| Revascularization | **<.0001** | **0.0236** | **0.674** | **0.478– 0.948** |
| **Medication at discharge** |  |  |  |  |
| Aspirin | **<.0001** | **<.0001** | **0.210** | **0.116– 0.379** |
| Other antiplatelets | **<.0001** | **0.0372** | **0.664** | **0.451– 0.976** |
| Beta blockers | **<.0001** | **0.0199** | **0.665** | **0.472– 0.937** |
| Statin | **<.0001** | **<.0001** | **0.213** | **0.124– 0.364** |
| **Complications** |  |  |  |  |
| In-hospital heart failure | **<.0001** | **0.0010** | **1.910** | **1.299– 2.810** |
| Recurrent MI (In Hospital Infarction/Re-Infarction) | <.0001 | 0.8767 | 0.951 | 0.503– 1.796 |
| Stroke | <.0001 | 0.4856 | 1.494 | 0.483– 4.621 |
| Major Bleeding | <.0001 | 0.8130 | 0.840 | 0.197– 3.571 |
| Atrial Fibrillation | <.0001 | 0.1991 | 0.473 | 0.151– 1.483 |
| OHCA | <0.001 | 0.8393 |  |  |
| Young | <.0001 | 0.4562 |  |  |
| **Interaction Terms*** |  | 0.6394 |  |  |
| OHCA in Young adults |  |  | 1.547 | 0.246– 9.744 |
| OHCA in Older adults |  |  | 0.816 | 0.114– 5.823 |
| Young adults with OHCA |  |  | 0.363 | 0.025– 5.214 |
| Young adults with no OHCA |  |  | 0.689 | 0.493– 0.961 |

OR: Odds ratio. CI: Confidence interval. MI: Myocardial infarction. STEMI: ST elevation myocardial infarction. LV: Left Ventricle. UH: Unfractionated heparin. LWMH: Low molecular weight heparin. OHCA: Out of hospital cardiac arrest.

*Interaction analysis was performed for the variables “young age” and “OHCA” (Young*OHCA).

**S5C Table: Comparison of mortality rates of OHCA patients before and after 2011.**

| **Variables** | **Before 2011** | **After 2011** | **Total** | **P value** |
| --- | --- | --- | --- | --- |
| **Overall OHCA patients** |  |  |  |  |
| In-hospital mortality | 114/262  (43.51%) | 151/349  (43.27%) | 265/611  (43.37%) | 0.9518 |
| One month mortality | 31/49  (63.27%) | 98/179  (54.75%) | 129/228  (56.58%) | 0.2865 |
| One year mortality | 33/47  (70.21%) | 95/173  (54.91%) | 128/220  (58.18%) | 0.0593 |

OHCA: Out of hospital cardiac arrest.
