# Supplementary material for: Characteristics and predictors of out-of-hospital cardiac arrest in young adults hospitalized with acute coronary syndrome: A retrospective cohort study of 30,000 patients in the Gulf region
Source: PLoS One. 2023 May 25;18(5):e0286084. doi: 10.1371/journal.pone.0286084 (PMC10212072; doi:10.1371/journal.pone.0286084)
Supplement: Attachment — Submitted filename: Response to Reviewers.docx [file pone.0286084.s007.docx]

May 8, 2023

RE: **“Characteristics and predictors of out-of-hospital cardiac arrest in young adults hospitalized with acute coronary syndrome: A retrospective cohort study of 30,000 patients in the Gulf region.”** (PONE-D-22-33714R1)

Dear Dr. AlSaif,

The authors would like to thank the editor and reviewers again for reviewing the revised manuscript. We have updated the manuscript based on the reviewers’ suggestions. The comments are in bold, and our responses follow.

**Reviewer #1:**

**The authors are thanked for their consideration, revisions and additional analyses. To the reviewer’s opinion, the adjustments that are made benefit the manuscript. The manuscript is suitable for publication, but the reviewer recommends to consider the request addressed below.

Although the conclusion that OHCA is a sentinel event of CAD appears reasonable from a clinical perspective, the reviewer remains hesitant about the data on which this conclusion is drawn. Is it possible that the missing data introduce a bias for this variable? The authors are asked to provide an explanation for the missing data. Specifically, the authors are asked why this variable is only available for 41 out of the 188 OHCA patients, and how this may impact the obtained results. Furthermore, the authors are requested to include this in the limitations section and to attenuate the concluding statement of this finding.**

The reason for the “missing data” is that this variable was only available in some of the registries that contributed to the data set. It is certainly possible that this may have introduced a selection bias. We have added this explanation, discuss it in the limitation section, and revised our conclusion:

- Page 23, Line 268: added “It should be noted that this finding is limited by the smaller sample size available for this variable, given that it was not collected in all registries.”
- Page 29, Line 287: added “Fourth, our finding of OHCA as the sentinel event of CAD was based on a smaller sample compared to the other variables, which affects the precision of this estimate.”
- Page 30, Line 298: modified the conclusion sentence to “Moreover, OHCA was the sentinel event of CAD in many young adults.”

**Reviewer #2:**

**the authors have appropriately addressed most of my concerns. I would only like to see a direct comparison of the time-dependent analysis based on the two time-periods with regards to mortality in one single table. This reviewer believes that outcomes are substantially dependent on treatment modalities and these differ quite substantially among time-periods chosen (20.7% vs. 16.5%).**

We thank the reviewer for their valuable feedback. A new supplemental table for the results of time dependent mortality rates between the two time periods was added as suggested (Table S5C). Also, we added a summary of the results of this table in the results section of the manuscript.

- Added S5C Table as a new supplemental information file.
- Page 21, Line 210: added “S5C Table compares the mortality rates of OHCA patients before and after 2011. We observed higher mortality before 2011, albeit not statistically significant.”

**Journal Requirements:

Please review your reference list to ensure that it is complete and correct. If you have cited papers that have been retracted, please include the rationale for doing so in the manuscript text, or remove these references and replace them with relevant current references. Any changes to the reference list should be mentioned in the rebuttal letter that accompanies your revised manuscript. If you need to cite a retracted article, indicate the article’s retracted status in the References list and also include a citation and full reference for the retraction notice.**

We have reviewed the references and ensured that it is complete and correct.
